# Association of omega-3 fatty acids (EPA, DHA, DPA) and vitamin D with mortality and morbidity outcomes in COVID-19 patients: the COMED study

**DOI:** 10.3389/fnut.2026.1892752

**Published:** 2026-07-20

**Authors:** Svatava Bischofová, Zuzana Měřínská, Miroslava Beňovská, Petr Husa, Irena Řehůřková, Jana Klinerová, Ludmila Bourková, Klára Horáková, Štěpánka Dvořáková, Alice Hoffmannová, Martina Podborská, Jiří Ruprich

**Affiliations:** 1 National Institute of Public Health, Centre for Health, Nutrition and Food, Brno, Czechia; 2 Department of Laboratory Medicine, University Hospital Brno, Brno, Czechia; 3 Department of Laboratory Methods, Faculty of Medicine, Masaryk University, Brno, Czechia; 4 Department of Infectious Diseases, University Hospital Brno, Brno, Czechia; 5 Faculty of Medicine, Masaryk University, Brno, Czechia; 6 Department of Haematology, University Hospital Brno, Brno, Czechia; 7 Centre for Laboratory Medicine, Trauma Hospital Brno, Brno, Czechia; 8 Department of Laboratory Medicine, Masaryk Memorial Cancer Institute, Brno, Czechia

**Keywords:** COVID-19, disease severity, hospitalization, modified omega-3 index, mortality, omega-3 fatty acids, vitamin D

## Abstract

**Background and aims:**

Long-chain omega-3 fatty acids (FAs) and vitamin D (VitD) are implicated in the modulation of immune responses and inflammatory processes within the body. The COMED study evaluated levels of DHA, EPA and DPA (expressed as a modified omega-3 index, mO3I, defined as the percentage of these FAs relative to total FAs in whole blood) and VitD in patients with COVID-19 in relation to mortality and severe disease, as well as additional morbidity-related outcomes, including probability of hospitalisation and length of hospital stay (LOS).

**Methods:**

A total of 160 patients were enrolled, of whom 28 died during follow-up. Associations between mO3I and VitD levels and clinical outcomes were analysed using logistic and linear regression models adjusted for age, sex, BMI, smoking, diabetes mellitus, and hypertension.

**Results:**

Higher mO3I levels (Q4 ≥ 6.39%) were associated with lower odds of death (OR = 0.24, 95% CI: 0.05–0.87) and lower probability of hospitalisation (OR = 0.28, 95% CI: 0.09–0.91) compared with lower mO3I levels (Q1–Q3). Higher VitD levels (Q4 ≥ 71.9 nmol/L) were associated with a shorter LOS by 2.19 days compared with Q1–Q3, while higher levels (Q2–Q4 > 36.7 nmol/L) shortened LOS by 2.96 days compared with Q1. Age was the strongest predictor of mortality, severe disease, and LOS; BMI increased the probability of severe disease; smoking prolonged LOS.

**Conclusion:**

Higher mO3I levels were associated with lower odds of death, with similar trends observed for hospitalisation, whereas higher VitD levels were associated with a shorter LOS. Nutritional biomarkers and modifiable lifestyle factors, such as body weight and smoking, were associated with differences in clinical outcomes in COVID-19. However, the observational design of the study precludes causal interpretations.

## 1 Introduction

At the end of 2019, the first cases of pneumonia caused by the novel coronavirus SARS-CoV-2, the aetiological agent of COVID-19, were reported in the Chinese city of Wuhan (1). The pandemic was declared by the World Health Organization (WHO) on 11 March 2020 and lasted until 5 May 2023 (2, 3). Over this period, the disease caused more than 6.7 million deaths globally (4). COVID-19 was characterised by a wide spectrum of clinical symptoms—from mild fatigue to life-threatening pneumonia (5, 6). In the Czech Republic, this infection became the second leading cause of death in 2020 (8%) and even the leading cause in 2021 (18% of all deaths). Most deaths occurred in hospital among patients with severe disease. The most common comorbidities were hypertension, diabetes, ischaemic heart disease and malignancy, although approximately one fifth of the deceased had no underlying conditions. The mean age of those who died was 79 years, with an overall age range of 40–84 years (7, 8).

Acute inflammation is a key defence mechanism mediated by immune cells and chemical mediators (9). Severe COVID-19 is not determined by viral load alone, but primarily by a dysregulated immune response with insufficient resolution of inflammation (10, 11). This condition is characterised by pulmonary hyperinflammation and a cytokine storm associated with the massive release of pro-inflammatory cytokines (TNF-α, IL-6, IL-1, IL-8, MCP-1, etc.), which can lead to acute respiratory distress syndrome and multiorgan failure (12–14). Inflammation is further amplified by the death of infected cells and by released cellular debris, which activate inflammasomes and trigger the eicosanoid storm—the overproduction of pro-inflammatory lipid mediators such as prostaglandins (PGs) and leukotrienes (12, 15). Certain early eicosanoids (e.g., PGE₂) also initiate the biosynthesis of specialised pro-resolving mediators (SPMs), which play a key role in the termination of inflammation. SPMs promote the clearance of cellular debris, suppress the release of pro-inflammatory cytokines and restore tissue homeostasis. SPMs are biosynthesised from omega-6 and omega-3 polyunsaturated FAs (11, 12), including lipoxins from arachidonic acid (AA), and resolvins, protectins and maresins from docosahexaenoic acid (DHA), eicosapentaenoic acid (EPA) and docosapentaenoic acid (DPA). Studies show that patients with COVID-19 exhibit significantly higher concentrations of pro-inflammatory lipids derived from omega-6 FAs than SARS-CoV-2 negative individuals, whereas recovered patients display increased expression of SPMs. In severe cases, markedly reduced plasma concentrations of SPMs have been reported, along with increased concentrations of pro-inflammatory lipids derived from AA, reduced expression of enzymes involved in SPM biosynthesis, and lower expression of their receptors on circulating leukocytes (9, 11, 16).

VitD is known for its immunomodulatory properties and its ability to influence the balance between pro-inflammatory and anti-inflammatory mediators. In individuals with COVID-19, low VitD levels have been associated with increased concentrations of inflammatory markers such as IL-6, CRP and D-dimers (17, 18). VitD deficiency has been in several studies associated with an increased risk of infection, a higher probability of hospitalisation and increased mortality (19, 20).

The above findings suggest that omega-3 FAs and VitD may be involved in the modulation of the immune response and supporting the resolution of inflammation.

During 2021, the COMED study (COVID-19, OMEga-3, vitamin D) was conducted in collaboration with the University Hospital Brno (FN Brno). The aim was to analyse the associations between levels of omega-3 FAs (EPA, DHA, DPA), VitD and clinical outcomes in patients with COVID-19. The primary outcomes were mortality and severe disease (defined as the need for respiratory support and admission to the intensive care unit, ICU), while probability of hospitalisation and LOS were analysed as additional outcomes reflecting different aspects of morbidity. We hypothesised that higher mO3I (including EPA, DHA and DPA) and higher VitD levels would be associated with lower odds of death and severe disease. Associations with hospitalisation and LOS were also explored as additional indicators of morbidity.

## 2 Materials and methods

### 2.1 Participant selection and sample characteristics

Adults with confirmed COVID-19 infection (PCR or antigen test) having presented to the FN Brno, the second largest hospital in the Czech Republic, between February and May 2021 were initially screened by the clinical team. The inclusion criteria were a newly diagnosed COVID-19 infection as the primary diagnosis, respiratory symptoms consistent with SARS-CoV-2 pneumonia, and an elevated CRP > 5 mg/L.

A total of 217 patients were identified during preliminary screening. After applying the inclusion criteria, the final cohort consisted of 160 participants. The minimum required sample size was 110 based on estimated incidence rates and statistical power (80%; *α* = 0.05).

Of the 160 participants, 148 were hospitalised and 12 treated on an outpatient basis. The cohort consisted of 91 men and 69 women, with a mean age of 64 years, and 28 deaths occurred.

The severity of COVID-19 was assessed using a modified WHO classification and the recommended procedures applied in hospital practice. Patients were categorised into five grades (I–V), corresponding to three clinical categories: mild (grade I—asymptomatic; grade II—mild symptoms without the need for oxygen therapy), moderate (grade III—requiring oxygen therapy and standard hospitalisation), and severe, requiring intensive care (grade IV—requiring high-flow nasal oxygen therapy or non-invasive ventilation; grade V—requiring invasive mechanical ventilation or extracorporeal membrane oxygenation) (21–23). Disease severity was assessed at hospital presentation and at the worst recorded clinical point during hospitalisation.

The study was conducted in accordance with the Declaration of Helsinki and was reviewed by the Ethics Committee of the National Institute of Public Health (NIPH): ref. no. SZÚ/01773/2021; 8 February 2021. Biological blood samples were provided for scientific and research purposes in accordance with Section 81(4) of Act No. 372/2011 Coll., on Health Services. Pseudonymised clinical data were provided without direct identifiers, and the analytical team had no access to information enabling patient identification. As only pseudonymised, routinely collected clinical data and legally provided biological samples were used, individual informed consent was not required. All study outputs are reported exclusively in aggregated form, with no possibility of identifying individual patients.

### 2.2 Variables and data sources

The main independent variables of interest were mO3I and VitD levels. The mO3I was defined in COMED study as the sum of EPA, DHA and DPA, expressed as a percentage of total analysed FAs in whole blood.

The dependent variables were mortality and morbidity-related outcomes: severe disease, probability of hospitalisation, and LOS.

Selected demographic and clinical characteristics were included in the analyses, such as age, sex, BMI, smoking status, and the presence of comorbidities (hypertension and diabetes mellitus, DM). These variables were selected *a priori* based on their known associations with COVID-19 severity and clinical outcomes.

All data, except for the blood analyses of FAs and VitD, were obtained from pseudonymised medical records, including clinical laboratory values recorded at hospital presentation.

### 2.3 Laboratory analysis

Blood samples were collected on the first day of hospital presentation. Whole blood collected in K₃EDTA tubes was used for the FAs analysis, and corresponding serum samples for the VitD analysis (25-hydroxyvitamin D₃ and 25-hydroxyvitamin D₂).

VitD levels were determined on the sampling day at FN Brno using an automated immunoassay on the e801 module of the cobas 8000 system (F. Hoffmann-La Roche Ltd.), employing the electrochemiluminescent competitive immunoassay Elecsys Vitamin D Total II (Ref. 07028148 190). The laboratory is accredited according to ISO 15189:2012.

The whole noncoagulated blood was stored at −70 °C until analysed at the NIPH. The determination of FAs was based on their esterification with 13% boron trifluoride (BF₃) in methanol by heating in a water bath (80 °C, 1 h). The resulting fatty acid methyl esters (FAMEs) were extracted into hexane and analysed by GC-FID (Trace 1310/FID system; Thermo Scientific) with a ZB FAME capillary column (Phenomenex; 60 m × 0.25 mm × 0.2 um). Identification was carried out by the reference standard 673B (Nu Chek Prep, Inc.), corresponding to the erythrocyte FAs composition. Quantification was performed using an external standard method based on chromatographic peak areas. Method accuracy was verified using the 37 Component FAME Mix (Supelco).

The FAs content was expressed as the percentage of individual FAs relative to the sum of all analysed FAMEs. The method was validated in the NIPH laboratory accredited to ISO/IEC 17025:2018. In total, 20 FAs were analysed, from which the mO3I was calculated by the interpreters.

### 2.4 Statistical analyses

Continuous and categorical variables were summarised using standard descriptive methods.

Differences between severity groups (mild, moderate, and severe) were assessed using the Kruskal–Wallis test with *post-hoc* Dunn’s test and Bonferroni correction; for categorical variables, the Fisher’s exact test with Bonferroni correction was applied. For comparisons between survivors and non-survivors, the Wilcoxon test was used for continuous variables, and the chi-square test or Fisher’s exact test was used for categorical variables.

Data analysis was conducted in three phases. First, intercorrelations were assessed using Spearman’s coefficient to exclude multicollinearity. Second, univariate regression models were constructed for each independent variable (mO3I, VitD, the combination of mO3I + VitD, age, sex, BMI, smoking status, diabetes mellitus, hypertension) in relation to each clinical outcome, using the enter method. Finally, quartile analyses of mO3I and VitD were conducted across three models: unadjusted, adjusted for age and sex, and adjusted for all covariates (age, sex, BMI, smoking status, diabetes—DM, and hypertension). Quartile-based analyses were pre-specified, while subsequent grouping of quartiles was performed in an exploratory, data-driven manner. In the analyses of individual quartiles, Q1 served as the reference quartile, whereas in the analyses of combined quartiles, Q1–Q3 served as the reference group when compared with Q4, or Q1 when compared with the combined group Q2–Q4. Associations between mO3I and VitD levels and the binary clinical outcomes were assessed using logistic regression with Firth’s penalised logistic regression for low event outcomes. LOS was analysed using linear regression (*β*). In regression analyses, disease severity was defined as the worst recorded clinical status during the course of hospitalisation. The results are presented as odds ratios (OR) or *β* with 95% confidence intervals (CI) and *p* values, with statistical significance set at *p* < 0.05.

Mortality and severe disease were considered the primary outcomes, whereas hospitalisation and LOS were analysed as additional outcomes.

All analyses were performed in R (version 4.4.3).

## 3 Results

### 3.1 Disease severity at hospital presentation, in-hospital disease course, and survival status

When comparing the groups by disease severity at hospital presentation, patients with mild disease were, on average, younger, had a lower BMI, a lower prevalence of comorbidities (DM and hypertension), higher mO3I and VitD levels, and lower clinical parameter values than patients with moderate or severe disease. However, most laboratory parameters in the mild group still exceeded the upper limits of their respective reference ranges. With increasing severity, inflammatory markers, and liver enzymes progressively deteriorated, whereas levels of factors associated with more favourable outcomes (mO3I, VitD) declined. Characterisation of individuals according to survival status showed that survivors were, on average, younger, had a lower BMI, a lower prevalence of comorbidities, higher mO3I and VitD levels, and lower levels of inflammatory markers and liver enzymes compared with non-survivors. The LOS was, on average, 2.3 days shorter in survivors. Details are presented in Table 1.

**Table 1:** Characteristics of COVID-19 patients by disease severity at hospital presentation and survival status.

Parameter	Classification of disease severity at hospital presentation			Survival status	
	Count (*n* or %) or mean ± SD			Count (*n* or %), or mean ± SD	
	Mild disease (grade II)^a^	Moderate disease (grade III)^b^	Severe disease (grades IV and V)^c^	Survivors (82.5%)	Non-survivors (17.5%)
n (M/F)	57 (31/26)	93 (53/40)	10 (7/3)	132 (75/57)	28 (16/12)
Age*^ab^	56.9 ± 14.3*	68.0 ± 13.1*	62.5 ± 17.4	61.5 ± 14.6*	74.0 ± 10.3*
LOS*^ab^	6.7 ± 5.4*	12.8 ± 6.0*	10.8 ± 5.8	10.12 ± 6.1	12.4 ± 7.8
BMI (kg/m^2^)	28.9^↑^ ± 4.5	29.9^↑^ ± 6.1	31.7^↑^ ± 8.4	29.6^↑^ ± 5.6	29.7^↑^ ± 6.6
Smoking (%)	7 (12%)	18 (19%)	0 (0%)	24 (18%)	1 (3.6%)
DM (%)	13 (23%)	29 (31%)	3 (30%)	36 (27%)	9 (32%)
Hypertension (%)*^ab^	19 (33%)*	55 (59%)*	7 (70%)	63 (48%)	18 (64%)
mO3I (%)*^ac^	5.9 ± 1.3*	5.6 ± 1.2	5.0 ± 1.0*	5.8 ± 1.3	5.4 ± 1.0
VitD (nmol/L)	57.4^↓^ ± 20.2	55.5^↓^ ± 27.7	44.6^↓^ ± 21.5	56.3^↓^ ± 23.6	51.7^↓^ ± 30.7
CRP (mg/L)*^ab, ac, bc^	45.9^↑^ ± 35.2*	81.6^↑^ ± 60.2*	141.3^↑^ ± 43.7*	68.3^↑^ ± 56.9*	92.5^↑^ ± 53.0*
WBC (10^9/L)^*ac, bc^	6.02 ± 2.1*	6.97 ± 3.5*	8.84 ± 2.3*	6.57 ± 2.76	7.56 ± 4.1
NEU (relative count)*^ab, ac, bc^	0.71 ± 0.13*	0.77^↑^ ± 0.10*	0.85^↑^ ± 0.10*	0.74^↑^ ± 0.12*	0.81^↑^ ± 0.10*
NLR ratio*^ab, ac, bc^	4.4^↑^ ± 2.6*	8.4^↑^ ± 8.4*	10.8^↑^ ± 5.1*	6.2^↑^ ± 4.9*	11.7^↑^ ± 12.1*
D-dimers (mg/L)*^ab, ac^	1.19^↑^ ± 2.6*	2.13^↑^ ± 3.1*	3.16^↑^ ± 2.6*	1.48^↑^ ± 2.6*	3.63^↑^ ± 3.9*
ALT (μkat/L)	0.77 ± 0.7	1.07^↑^ ± 1.5	3.01^↑^ ± 6.3	0.99^↑^ ± 1.4	1.54^↑^ ± 3.8
AST (μkat/L)*^ab, ac^	0.88^↑^ ± 0.5	1.21^↑^ ± 0.7	5.46^↑^ ± 12.6	1.06^↑^ ± 0.7*	2.74^↑^ ± 7.6*

Reference ranges of clinical parameters: VitD: 75–200 nmol/L; CRP: 0–5 mg/L; WBC: 4–10 × 10^9^/L; NEU (relative count): 0.47–0.70; NLR ratio: <4; D-dimers: 0–0.5 mg/L; ALT: men 0.17–0.83 μkat/L, women 0.25–0.58 μkat/L; AST: men 0.17–0.85 μkat/L; women <0.17–0.60 μkat/L; BMI: 18.5–25 kg/m^2^.

^*^Indicates statistical significance; superscript “a, b, c” indicate pairs of groups between with significant differences (*p* < 0.05); ^↑/↓^denote values outside the reference ranges; M, male; F, female; LOS, length of hospital stay.

The dynamics of clinical status during hospitalisation showed deterioration in one fifth of patients. A severe disease was experienced by 20% of patients, and this category also exhibited the highest mortality. Among patients admitted with mild disease, only one patient died; this individual progressed to severe disease during the hospital stay. Details are presented in Table 2.

**Table 2:** Dynamics of clinical condition in COVID-19 patients: comparison of status at presentation and worst recorded status.

Admission status			Worst recorded status compared to admission status				*n* deteriorated vs. admission status	*n* † in the respective grade (%)
Clinical status	Severity grade	Number of patients (%)	Grade II *n* (*n* †)	Grade III *n* (*n* †)	Grade IV *n* (*n* †)	Grade V *n* (*n* †)		
Mild disease	II	57 (36%)	46 (0)	9 (0)	1 (0)	1 (1)	11	1 (2%)
Moderate disease	III	93 (58%)	–	72 (7)	9 (3)	12 (11)	21	21 (22%)
Severe disease	IV	8 (5%)	–	–	5 (1)	3 (3)	3	4 (50%)
	V	2 (1%)	–	–	–	2 (2)	0	2 (100%)

†, non‑survivors.

### 3.2 Basic analysis of relationships between variables

Spearman’s correlation analysis did not reveal any strong correlations indicating a risk of multicollinearity.

In the univariate, unadjusted regression models, no statistically significant associations were observed for mO3I, VitD, or their combined indicator (mO3I + VitD) with any of the assessed clinical outcomes. For this reason, the individual biomarkers were subsequently evaluated separately using quartile-based analyses in both unadjusted and adjusted models.

### 3.3 Modified omega-3 index: quartile analysis (unadjusted and adjusted models)

Table 3 presents the cohort stratified into quartiles together with selected demographic characteristics and deaths.

**Table 3:** Demographic characteristics and mortality by mO3I quartiles in COVID-19 patients.

Quartile	mO3I (%): mean ± SD (min–max)	Age (years): mean ± SD	Sex-M % (*x*/*n*)	† % (*x*/*n*)
Q1	4.31 ± 0.51 (3.06–4.90)	62.2 ± 18.4	52.5% (21/40)	17.5% (7/40)
Q2	5.26 ± 0.18 (4.91–5.51)	62.0 ± 15.7	55% (22/40)	20.0% (8/40)
Q3	5.91 ± 0.26 (5.52–6.38)	66.8 ± 13.3	62.5% (25/40)	27.5% (11/40)
Q4	7.31 ± 0.99 (6.39–10.50)	63.9 ± 10.2	57.5% (23/40)	5.0% (2/40)

M, men; †, non-survivors, values are presented as percentage (number of non-survivors/total number in quartile).

No significant differences were observed when comparing all quartiles with clinical outcomes, so Q1–Q3 were subsequently combined and compared with Q4 in an exploratory analysis. As these analyses were exploratory, this grouping was used to assess potential threshold effects at higher biomarker levels. The odds of death were lower among individuals in Q4, and this association remained significant in the adjusted models (Table 4). This observed association was based on 2 deaths in Q4 compared with 26 deaths in Q1–Q3. Age increased mortality risk, while BMI showed a borderline effect.

**Table 4:** Associations of mO3I (Q4 vs. Q1–Q3) with mortality and morbidity indicators in COVID-19 patients.

Clinical outcome	Unadjusted models				Adjusted models (age and sex)				Adjusted models (age, sex, BMI, smoking, DM, hypertension)			
	Quartile (*n* Q4/*n* Q1–Q3)	OR	95% CI	*p*	Quartile/Covariate	OR	95% CI	*p*		OR	95% CI	*p*
Mortality*					Full model: *p* < 0.001				Full model: *p* < 0.001			
	**Q4** (2/26)	**0.23**	0.05–0.76	**0.013**	**Q4**	**0.25**	0.05–0.84	**0.023**	**Q4**	**0.24**	0.05–0.87	**0.027**
					**Age**	**1.07**	1.04–1.11	**<0.001**	**Age**	**1.09**	1.04–1.14	**<0.001**
					Sex (M)	1.37	0.57–3.41	0.490	Sex (M)	1.46	0.59–3.74	0.418
									BMI	1.09	0.99–1.18	0.052
									DM	0.69	0.23–1.93	0.480
									Hypertension	0.83	0.30–2.23	0.702
									Smoking	0.35	0.04–1.57	0.189
Severe disease*					Full model: *p* = 0.26				Full model: *p* **< 0.05**			
	Q4 (7/26)	0.80	0.30–1.89	0.620	Q4	0.79	0.30–1.89	0.608	Q4	0.79	0.25–2.49	0.633
					Age	1.02	1.00–1.05	0.086	**Age**	**1.05**	1.01–1.09	**<0.01**
					Sex (M)	1.56	0.71–3.52	0.265	Sex (M)	1.67	0.73–3.92	0.224
									**BMI**	**1.12**	1.04–1.21	**<0.01**
									DM	0.45	0.16–1.15	0.092
									Hypertension	1.50	0.62–3.66	0.367
									Smoking	2.53	0.91–6.80	0.073
Hospitalisation*					Full model: *p* = 0.07				Full model: *p* = 0.305			
	Q4 (34/114)	0.30	0.09–0.97	0.045	**Q4**	**0.28**	0.08–0.93	**0.039**	**Q4**	**0.28**	0.09–0.91	**0.035**
					Age	1.04	1.00–1.08	0.083	Age	1.05	1.00–1.11	0.050
					Sex (M)	1.56	0.47–5.19	0.458	Sex (M)	1.70	0.51–5.84	0.387
									BMI	1.03	0.93–1.16	0.644
									DM	0.88	0.22–4.05	0.862
									Hypertension	0.53	0.12–2.10	0.369
									Smoking	1.86	0.4–17.76	0.467
LOS**					Full model: *p* **< 0.001**				Full model: *p* **< 0.001**			
		*β* (days)	95% CI	*p*		*β* (days)	95% CI		*p*	*β* (days)	95% CI	*p*
	Q4	−0.63	−2.96–1.70	0.592	Q4	−0.69	−2.88–1.51	0.537	Q4	−0.60	−2.79–1.59	0.588
					**Age**	**0.15**	0.09–0.22	**<0.001**	**Age**	**0.18**	0.01–0.25	**<0.001**
					Sex (M)	1.59	−0.35–3.53	0.108	Sex (M)	1.63	−0.34–3.60	0.105
									BMI	0.10	−0.09–0.28	0.307
									DM	−1.59	−3.89–0.71	0.173
									Hypertension	0.65	−1.53–2.83	0.556
									**Smoking**	**2.64**	0.01–5.23	**0.049**

OR, odds ratio; CI, confidence interval (95%); *p*-value (test of statistical significance); *β*, linear regression coefficient, representing the change in length of hospital stay (in days); LOS, length of hospital stay; *Firth’s penalised likelihood model for small sample sizes; **linear regression; bold type, statistically significant result; M, male; DM, diabetes mellitus. *In the Hospitalisation section (unadjusted model), Q4, OR = 0.30 and *p* = 0.045 should be shown in bold as statistically significant results.

The odds of severe disease were lower in Q4, although not significantly. Age and BMI were significant predictors of severe disease.

The odds of hospitalisation were significantly lower in Q4 compared with Q1–Q3 and this association remained significant after adjustment, although the overall model was not statistically significant. No other factor, except for the borderline effect of age, was significant.

LOS was comparable between Q4 (10 ± 7.78 days) and Q1–Q3 (10.7 ± 5.96 days), with no significant difference found. In contrast, age and smoking were significant predictors of prolonged LOS.

### 3.4 Vitamin D: quartile analysis (unadjusted and adjusted models)

Table 5 shows the cohort stratified into VitD quartiles with selected demographic characteristics and deaths.

**Table 5:** Demographic characteristics and mortality by VitD quartiles in COVID-19 patients.

Quartile	VitD (nmol/L): Mean ± SD (min–max)	Age (years) Mean ± SD	Sex-M % (*x*/*n*)	† % (*x*/*n*)
Q1	25.6 ± 7.07 (12.4–36.7)	68.8 ± 14.3	52.5% (21/40)	30% (12/40)
Q2	45.9 ± 5.06 (36.7–53.9)	60.2 ± 16.5	67.5% (27/40)	12.5% (5/40)
Q3	62.4 ± 4.93 (54.2–71.1)	60.2 ± 12.9	47.5% (19/40)	12.5% (5/40)
Q4	88.2 ± 16.7 (71.9–147)	65.6 ± 13.9	60% (24/40)	15% (6/40)

M, male; †, non-survivors.

Testing for differences between the VitD quartiles and the dependent variables did not yield significant results, or any initial associations were attenuated after adjustment. Therefore, two additional exploratory comparisons were performed: Q4 vs. Q1–Q3, and Q2–Q4 vs. Q1, to explore potential threshold effects at both higher and lower VitD levels. Details of the selected analyses are presented in Table 6.

**Table 6:** Associations of VitD (Q4 vs. Q1–Q3 and/or Q2–Q4 vs. Q1) with mortality and morbidity indicators in COVID-19 patients.

Clinical outcome	Unadjusted models				Adjusted models (age and sex)				Adjusted models (age, sex, BMI, smoking, DM, hypertension)			
	Quartile (*n* Q4/*n* Q1–Q3, or *n* Q2–Q4/*n* Q1)	OR	95% CI	*p*	Quartile/Covariate	OR	95% CI	*p*		OR	95% CI	*p*
Mortality*					Full model: *p* **< 0.001**				Full model: *p* **< 0.001**			
	Q4 (6/22)	0.83	0.29–2.05	0.690	Q4	0.700	0.24–1.84	0.481	Q4	0.76	0.25–2.07	0.597
					**Age**	**1.078**	**1.04–1.12**	**<0.001**	**Age**	**1.09**	**1.04–1.14**	**<0.001**
					**Sex (M)**	**1.290**	**0.55–3.13**	**0.567**	Sex (M)	**1.37**	**0.56–3.41**	**0.494**
									BMI	1.08	0.99–1.65	0.072
									DM	0.60	0.20–1.64	0.323
									Hypertension	1.04	0.39–2.75	0.944
									Smoking	0.33	0.03–1.44	0.152
	Q2–Q4 (16/12)	**0.36**	0.15–0.84	**0.020**	Q2–Q4	0.502	0.21–1.25	0.14	Q2–Q4	0.53	0.21–1.34	0.177
					**Age**	**1.072**	**1.03–1.12**	**<0.001**	**Age**	**1.09**	**1.04–1.15**	**<0.001**
					**Sex (M)**	**1.310**	**0.55–3.19**	**0.55**	**Sex (M)**	**1.39**	**0.57–3.49**	**0.470**
									BMI	1.08	0.99–1.17	0.073
									DM	0.58	0.20–1.56	0.292
									Hypertension	1.03	0.40–2.72	0.944
									Smoking	0.36	0.04–1.58	0.196
Severe disease*					Full model: *p* = 0.26				Full model: *p* **< 0.01**			
	Q2–Q4 (23/10)	0.70	0.30–1.66	0.407	Q2–Q4	0.79	0.34–1.92	0.59	Q2–Q4	0.84	0.35–2.12	0.704
					**Age**	**1.02**	**1.00–1.05**	**0.11**	**Age**	**1.05**	**1.01–1.09**	**0.012**
					**Sex (M)**	**1.56**	**0.72–3.52**	**0.26**	**Sex (M)**	**1.68**	**0.74–3.95**	**0.220**
									**BMI**	1.12	1.04–1.21	**<0.010**
									DM	0.44	0.16–1.11	0.082
									Hypertension	1.53	0.64–3.75	0.340
									Smoking	2.54	0.92–6.86	0.073
Hospitalisation*					Full model: *p* = 0.283				Full model: *p* = 0.68			
	Q2–Q4 (109/39)	0.36	0.04–1.60	0.200	Q2–Q4	0.43	0.05–1.95	0.300	Q2–Q4	0.43	0.05–2.03	0.317
					Age	1.03	0.98–1.07	0.170	Age	1.04	0.99–1.09	0.106
					Sex (M)	1.58	0.48–5.23	0.438	Sex (M)	1.71	0.51–5.81	0.380
									BMI	1.02	0.91–1.16	0.770
									DM	0.70	0.17–3.24	0.624
									Hypertension	0.61	0.14–2.46	0.490
									Smoking	1.74	0.38–16.60	0.510
			
LOS**					Full model: *p* **< 0.001**				Full model: *p* **< 0.001**			
	Quartile	*β* (days)	95% CI	*p*		*β* (days)	95% CI	*p*		*β* (days)	95% CI	*p*
	Q4	−1.57	−3.88–0.75	0.184	Q4	−2.04	−4.22–0.14	0.0663	**Q4**	**−2.19**	−4.36–-0.013	**0.049**
					**Age**	**0.16**	0.09–0.22	**<0.001**	**Age**	**0.18**	0.104–0.26	**<0.001**
					Sex (M)	1.67	−0.25–3.59	0.088	Sex (M)	1.75	−0.21–3.70	0.080
									BMI	0.08	−0.10–0.26	0.360
									DM	−1.82	−4.10–0.45	0.115
									Hypertension	0.69	−1.45–2.83	0.526
									**Smoking**	**2.75**	0.150–3.34	**0.039**
	**Q2**–**Q4**	**−3.60**	−5.86–-1.34	**<0.002**	**Q2–Q4**	**−2.76**	−4.96–-0.56	**0.014**	**Q2–Q4**	**−2.96**	−5.17–-0.74	**<0.05**
					**Age**	**0.14**	0.07–0.20	**<0.001**	**Age**	**0.16**	0.08–0.2	**<0.001**
					Sex (M)	1.63	−0.27–3.54	0.093	Sex (M)	1.72	−0.21–3.65	0.080
									BMI	0.07	−0.11–0.25	0.464
									DM	−2.01	−4.27–0.25	0.082
									Hypertension	0.76	−1.36–2.88	0.481
									**Smoking**	**2.75**	0.17–5.32	**0.037**

OR, odds ratio; CI, confidence interval (95%); *p*-value (test of statistical significance); *β*, linear regression coefficient, representing the change in length of hospital stay (in days); LOS, length of hospital stay, *Firth’s penalised likelihood model for small sample sizes; **linear regression; bold type, statistically significant result; M, male; DM, diabetes mellitus.

The probability of death was lower in Q4 than in Q1–Q3, although not significantly. In the adjusted models, age remained the only significant predictor. Individuals in Q2–Q4 showed a significantly lower odds of death compared with Q1 before adjustment, but this effect lost significance after adjustment. Age remained the significant predictor of mortality.

For severe disease, individuals in Q2–Q4 had a lower risk compared with Q1, but the difference was not significant. In the fully adjusted model, age and BMI were significant predictors.

Higher VitD levels (Q2–Q4) were associated with a reduced probability of hospitalisation across all models, although not significantly.

LOS was the shortest in Q4 (9.35 ± 6.49 days) and the longest in Q1 (13.23 ± 6.36 days). A statistically longer hospital stay was found when comparing Q1 vs. Q2–Q4 (9.63 ± 6.24 days). After adjustment, Q4 significantly shortened LOS, whereas age and smoking prolonged it compared with Q1–Q3. Higher VitD levels (Q2–Q4) compared with Q1 also significantly shortened LOS. Age and smoking were the main predictors of LOS.

## 4 Discussion

Higher mO3I values (≥6.39%) were associated with more favourable clinical outcomes in the COMED study, particularly lower odds of death and hospitalisation, corresponding to approximately 76 and 72% lower odds, respectively. The mortality estimate, however, is based on a small number of events. Trends towards a reduced probability of severe disease and shorter LOS were consistent with a potentially favourable association, although not statistically significant. These associations may be partly explained by the anti-inflammatory and immunomodulatory properties of omega-3 FAs. DHA, EPA and DPA support the formation of SPMs, which facilitate the resolution of the inflammatory response and attenuate the consequences of a dysregulated immune reaction (9–16).

Evidence from international studies also suggests a protective association of higher omega-3 status, although the effect and the clinical endpoints vary across studies (24–31). A large cohort study from the UK Biobank (*n* = 466.572) demonstrated that regular fish oil supplementation was associated with a lower risk of SARS-CoV-2 infection (HR = 0.97, CI: 0.94–0.99), hospitalisation (HR = 0.92, CI: 0.85–0.98), and COVID-19 related mortality (HR = 0.86, CI: 0.75–0.98). Moreover, Mendelian randomisation has suggested a potential relationship between higher circulating DPA concentrations and a reduced risk of severe disease (OR = 0.26, CI: 0.08–0.88) (24). Harris et al. (25) showed that higher DHA concentrations were associated with lower risk of SARS CoV 2 infection (HR = 0.79, CI: 0.71–0.89) and hospitalisation (HR = 0.74, CI: 0.58–0.94), although no association was observed with mortality. Sun et al. (26) reported similar trends, with lower risk of SARS CoV 2 infection and severe disease in individuals with higher circulating levels of total omega-3 FAs and DHA. Mendelian randomisation analyses further suggested that higher DPA concentrations may be associated with a reduced risk of severe COVID-19 (OR = 0.89, CI: 0.81–0.99). The systematic review by Mazidimoradi et al. (27) concluded that omega-3 FAs reduce the risk of COVID-19 by 12–21%, and that low omega-3 levels are associated with higher risk of mechanical ventilation, hospitalisation, and mortality. Zapata et al. (28) confirmed that a higher O3I was associated with a lower risk of mechanical ventilation (OR = 0.46, CI: 0.21–1.00) and death (OR = 0.28, CI: 0.08–0.99). Asher et al. (29) reported a 75% lower odds ratio of death (OR = 0.25, *p* = 0.07) in individuals with an O3I ≥ 5.7%. In the case–control study by Ramírez-Santana et al. (30), a higher O3I was associated with a lower risk of severe disease (OR = 0.52, CI: 0.32–0.86), whereas DM (OR = 4.41, CI: 1.60–12.12) and age (OR = 1.03, CI: 1.002–1.062) increased the risk. Ramezani et al. (31) also suggested that higher omega-3 FAs intake was linked to lower inflammatory biomarkers (including CRP) and a reduced probability of severe disease (OR = 0.42, CI: 0.22–0.79).

A specific feature of the COMED study was the assessment of mO3I (the proportion of EPA, DHA and DPA relative to the sum of 20 FAs in whole blood). Preliminary analyses using a conventional omega-3 marker (EPA + DHA, analogous to the omega-3 index, O3I) showed no significant associations with mortality, either across individual or grouped quartiles. Therefore, subsequent analyses focused on mO3I as the primary omega-3-related exposure.

The O3I is most frequently described in the literature in relation to cardiovascular health, with values above 8% considered protective (32–35). The standard O3I is defined as the percentage of EPA and DHA in erythrocytes (34). In our study, FAs were measured in whole blood; thus, the calculated values represent a proxy measure and are not directly equivalent to the erythrocyte-based O3I; therefore, direct comparison with studies using the standard O3I should be approached with circumspection. In a sensitivity analysis using a conventional O3I (EPA + DHA alone based on whole blood) for mortality, the associations were in the same direction as for mO3I but weaker and not statistically significant after adjustment, which is consistent with the limited statistical power of the study and the small number of events.

In contrast to the standard O3I, mO3I also incorporates DPA—a metabolic intermediate with potential biological relevance. DPA plays a role in the metabolic pathway of DHA and EPA, and some studies, including Mendelian randomisation analyses, have suggested a potential association with COVID-19 severity (24, 26). In the COMED dataset, DPA accounts for an average of 1.4% (0.5–2.4%) of mO3I, corresponding to approximately one quarter (25%; 12.5–45.5%) of the index total. Thus, the inclusion of DPA may provide complementary information on omega-3 FAs status. However, the use of mO3I limits direct comparability with studies based on the conventional O3I.

To better contextualise these findings, we compared the COMED dataset with data from a non-randomised adult Czech population (*n* = 574, mean age 44 years), in which FAs were analysed using the dried (capillary) blood spot (DBS) method, with a mean mO3I of 4.7% (2.2–14.2%). An experimentally derived approach was applied to express COMED results on the DBS scale; however, this approach is based on experimental data and should be interpreted with caution. After conversion, mO3I values in COMED patients were 4.5% (2.5–8.2%) among survivors and 4.2% (2.6–5.7%) among non-survivors, slightly lower than those observed in the reference population.

The second independent variable evaluated in relation to mortality and morbidity in patients with COVID-19 was the VitD level (>36.7 nmol/L), which in the COMED study remained associated, even after adjustment, with a significant shorter LOS. This may be related to the well-established role of VitD in regulating both innate and adaptive immunity, which is known to influence the course of viral infections. The role of VitD is also supported by findings from other studies. The meta-analysis by Chiodini et al. (36) confirmed a consistent association between low VitD levels and poorer clinical outcomes of COVID-19—including a higher risk of infection, hospitalisation, ICU admission and death. Severe deficiency (<25 nmol/L), deficiency (<50 nmol/L) and insufficiency (<75 nmol/L) were associated with increased risks of ICU admission (OR = 2.63, CI: 1.45–4.77; OR = 2.16, CI: 1.43–3.26; OR = 2.83, CI: 1.74–4.61), death (OR = 2.60, CI: 1.93–3.49; OR = 1.84, CI: 1.26–2.69; OR = 4.15, CI: 1.76–9.77), infection (OR = 1.68, CI: 1.32–2.13; OR = 1.83, CI: 1.43–2.33; OR = 1.49, CI: 1.16–1.91) and hospitalisation (OR = 2.51, CI: 1.63–3.85; OR = 2.38, CI: 1.56–3.63; OR = 1.82, CI: 1.43–2.33) (36). Campi et al. (37) showed that patients with severe disease had markedly lower VitD levels (mean 45 nmol/L) than those with mild disease (76 nmol/L) and healthy controls (64 nmol/L). Patients admitted to the ICU or those who died had lower levels (36 nmol/L and 33 nmol/L, respectively) than the remaining hospitalised patients (56 nmol/L) and survivors (48 nmol/L). Low VitD levels correlated with higher IL 6 concentrations, supporting its role in the inflammatory response (37). Individuals in the COMED study admitted with mild disease had a mean VitD level of 57.4 nmol/L, those with moderate disease 55.5 nmol/L, and those with severe disease 44.6 nmol/L, consistent with the findings of Campi et al. (37). Similarly, Konikowska et al. (38) reported significantly lower VitD concentrations in non-survivors compared with survivors among patients with severe acute respiratory syndrome, with the risk of death increasing markedly at levels below 38.5 nmol/L. An Iranian prospective observational study (2021) likewise confirmed higher mortality in patients with VitD deficiency (<50 nmol/L), both prior to infection (OR = 2.01, *p* = 0.041) and at hospital admission (HR = 2.35, *p* = 0.019) (39). A large Indian prospective cohort study further demonstrated that low VitD levels were significantly associated with more severe disease and higher mortality, whereas higher VitD concentrations exerted a protective effect (AOR = 0.87, CI: 0.80–0.94) (40).

According to data from the Czech biomonitoring programme (2018), the mean VitD level in the adult Czech population is 60.8 nmol/L, falling to 51.41 nmol/L in winter and 53.45 nmol/L in spring (41), which corresponds to the period when COMED samples were collected. The mean VitD concentration in our study population is comparable to these population-based values; however, the Endocrine Society’s recommendation for disease prevention is >75 nmol/L (42), a threshold met by only 21% of patients in the COMED cohort.

The assessment of the combined effects of mO3I and VitD did not demonstrate any significant association with clinical outcomes, supporting the view that their effects are independent but complementary. While omega-3 FAs contribute to the resolution of inflammation and the restoration of tissue homeostasis, VitD influences the expression of genes involved in both innate and adaptive immunity. From a practical perspective, it is noteworthy that the levels of both biomarkers are readily modifiable through lifestyle—particularly diet. Lower omega-3 status in the Czech population is related to low consumption of fatty fish, which are their almost exclusive dietary source. According to the most recent data (2022), consumption of fisheries and aquaculture products in the Czech Republic is nearly fourfold lower than the EU average (5.9 kg vs. 23.5 kg live weight/person/year) (43). Low VitD levels are likewise attributable to low dietary intake, which is particularly important during periods when endogenous synthesis in the skin cannot be relied upon. According to NIPH data, the median dietary intake of VitD (excluding dietary supplements) in the Czech population aged over 4 years is <5.1 ug/person/day (44), represents at most one third of the EFSA recommendation and approximately one quarter of the D-A-CH recommendation (45, 46). From a public health perspective, attention should therefore focus on promoting foods rich in omega-3 FAs and VitD, and on considering food fortification or targeted supplementation in at risk groups.

The limitations of the COMED study lie in the relatively small number of patients, particularly after stratification into quartiles, which limits generalisability of the findings. This is further reflected in the relatively small number of events, as the observed association between mO3I and mortality is based on only 2 deaths in Q4 compared with 26 in Q1–Q3. Although appropriate statistical procedures, including Firth’s penalised regression and adjustment for key covariates, were employed to minimise potential bias, the resulting estimates may remain unstable. Small changes in the distribution of events across quartiles could materially affect the observed effect size.

It should also be noted that the calculated minimum sample size referred to the overall cohort, whereas the quartile-stratified analyses, which underpin the main findings, are based on smaller subgroup sizes and may therefore be underpowered.

It must also be considered that the biomarkers (mO3I, VitD and other clinical parameters) were measured and assessed at the time of hospital presentation; therefore, an influence of the acute phase of the disease cannot be excluded. This limitation is supported by evidence showing that circulating 25(OH)D concentrations may decline rapidly, during acute inflammatory states, including COVID-19, with significant decreases observed within the first 48 h of hospitalisation (47, 48). It is well established that circulating 25(OH)D concentrations may decrease as part of the acute-phase response, and acute illness is also associated with substantial alterations in lipid and FAs metabolism, which may affect circulating levels. Therefore, lower biomarker levels observed in patients with more severe disease may at least partly reflect a consequence of the disease process rather than a direct causal factor. Accordingly, these findings should be interpreted with caution and considered exploratory and hypothesis-generating rather than confirmatory.

Furthermore, while quartile-based analyses were pre-specified, grouping of quartiles was performed in an exploratory, data-driven manner after initial analyses did not show significant differences across all four quartiles. This approach may increase the risk of type I error. In this cohort, all patients presented with respiratory symptoms and confirmed COVID-19 pneumonia and were clinically evaluated for the need for hospital admission. Therefore, this outcome reflects a clinical decision-making threshold rather than a purely administrative event.

The interpretation of the results may also be affected by the temporal context of the study—the data originate from a period before widespread vaccination and before the circulation of milder viral variants, meaning that the current clinical relevance in terms of mortality or severe disease risk may therefore differ. Nevertheless, the biological mechanisms related to the effects of omega-3 FAs and VitD remain broadly relevant to viral infections.

Despite these limitations, the study provides additional insights into the potential role of omega-3 FAs, specifically mO3I, and VitD as markers associated with disease severity and mortality in COVID-19. The strengths of the study include the laboratory-verified measurement of biomarkers from blood samples, adjustment for demographic and clinical covariates, and the use of mO3I, which also incorporates DPA.

The findings may contribute to a better understanding of nutritional factors associated with the course of infectious diseases and are broadly consistent with recommendations suggesting that maintaining optimal VitD levels (>75 nmol/L) and an O3I around 8% may be associated with favourable health outcomes.

## 5 Conclusion

Higher mO3I levels were associated with lower odds of death, with similar findings observed for hospitalisation among patients with COVID-19, supporting a potential beneficial role of omega-3 FAs. In contrast, VitD did not show a significant association with mortality or severe disease; however, higher VitD levels were significantly associated with a shorter LOS. Age remained the strongest predictor of mortality, severe disease, and LOS, whereas BMI increased the probability of severe disease, and smoking prolonged LOS. These findings highlight the relevance of nutritional biomarkers in the context of severe infectious diseases. From a public health perspective, these data underpin the importance of promoting long-term optimal nutritional status—particularly adequate intake of omega 3 FAs (DHA, EPA and DPA) and VitD—as these factors may be relevant in the context of exposure to infectious agents. However, the magnitude of these findings may differ in the current context of vaccination and circulating viral variants.

## Data Availability

The data supporting the findings of this study contain sensitive patient information and are therefore not publicly available. Data may be obtained from the corresponding author upon reasonable request.
